# Facet-Controlled LiMn_2_O_4_/C as Deionization Electrode with Enhanced Stability and High Desalination Performance

**DOI:** 10.1007/s40820-022-00897-3

**Published:** 2022-08-23

**Authors:** Yuxin Jiang, Liyuan Chai, Dehe Zhang, Fangping Ouyang, Xiangyuan Zhou, Sikpaam I. Alhassan, Sailin Liu, Yingjie He, Lvji Yan, Haiying Wang, Wenchao Zhang

**Affiliations:** 1 School of Metallurgy and Environment, Central South University, Changsha, 410083 People’s Republic of China; 2 Chinese National Engineering Research Center for Control and Treatment of Heavy Metal Pollution, Changsha, 410083 People’s Republic of China; 3 Water Pollution Control Technology Key Lab of Hunan Province, Changsha, 410083 People’s Republic of China; 4 School of Physics and Electronics, Hunan Key Laboratory for Super-Microstructure and Ultrafast Process, and Hunan Key Laboratory of Nanophotonics and Devices, Central South University, Changsha, 410083 People’s Republic of China; 5 State Key Laboratory of Powder Metallurgy, and Powder Metallurgy Research Institute, Central South University, Changsha, 410083 People’s Republic of China; 6 School of Chemical Engineering and Advanced Materials, Faculty of Sciences, Engineering and Technology, The University of Adelaide, Adelaide, 5005 Australia

**Keywords:** Deionization electrode, Desalination, Lithium-ion battery

## Abstract

**Highlights:**

- First report of a lithium-ion battery cathode as a deionization electrode for desalination.
- A novel approach to suppress manganese dissolution by exposing the (111) facet is proposed.
- Excellent desalination performance by the LiMn_2_O_4_/C cathode. The material achieves an ultrahigh desalination capacity of 117.3 mg g^−1^ at 1.0 V and a longer cycle life (200 cycles without capacity decay) with minor manganese dissolution during the cycling test in 10 mM aqueous LiCl solution.

**Abstract:**

Battery materials as emerging capacitive deionization electrodes for desalination have better salt removal capacities than traditional carbon-based materials. LiMn_2_O_4_, a widely used cathode material, is difficult to utilize as a deionization electrode due to its structural instability upon cycling and Mn dissolution in aqueous-based electrolytes. Herein, a facile and low-cost ball-milling routine was proposed to prepare a LiMn_2_O_4_ material with highly exposed (111) facets. The prepared electrode exhibited relatively low dissolution of Mn during cycling, which shows its long cycle stability. In the hybrid capacitive deionization system, the LiMn_2_O_4_/C electrode delivered a high desalination capacity of 117.3 mg g^−1^ without obvious capacity decay at a voltage of 1.0 V with a 20 mM initial salt concentration. In addition, the exposed (111) facets significantly alleviated Mn ion dissolution, which also enhanced the structural steadiness.
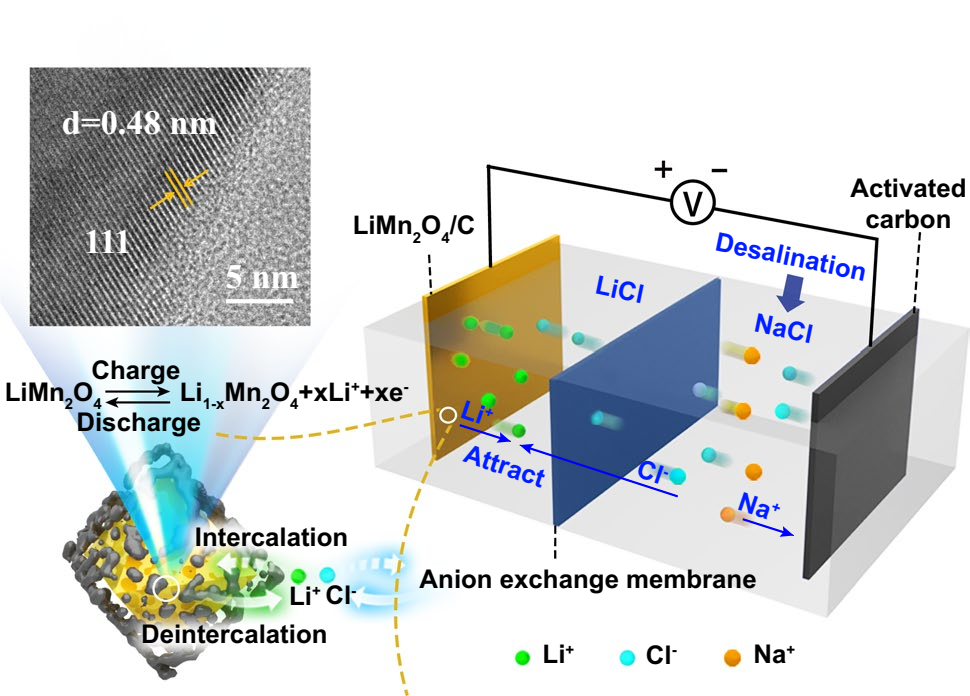

**Supplementary Information:**

The online version contains supplementary material available at 10.1007/s40820-022-00897-3.

## Introduction

The scarcity of freshwater is a global challenge, and desalination is a rational path toward alleviating the crisis because saltwater covers most of the surface of planet Earth [1–3]. Most of the current desalination techniques (such as reverse osmosis and distillation) work under high-pressure or high-temperature conditions, which are cost-intensive and not energy-efficient [4–6]. A green, affordable method with low energy consumption under mild operation conditions is in high demand. As a newly developed technology, capacitive deionization (CDI) could be a rational choice due to its cost-effectiveness and energy efficiency [7, 8]. A CDI cell provides an electric field that drives the salt ions to be adsorbed onto the electrode materials and simultaneously stores the energy. Traditional carbonaceous electrodes have low desalination capacities (< 15 mg g^−1^) because of the electrical double-layer (EDL) behavior of charge storage limited to the surface area [9, 10]. In the quest for higher deionization performance, battery electrodes have been employed in recent research owing to their larger electrochemical capacities, such as sodium-ion battery materials (sodium transition metal oxides, transition metal oxides, sodium super ion conductor materials, and Prussian blue analogues) and chloride battery materials (Ag-based or Bi-based materials, etc.) [11–19]. Although quite a few studies have initiated the use of battery materials applied in deionization systems, these materials are still difficult to scale up for real applications due to the low energy density and instability of the battery materials in aqueous-based electrolytes [20, 21].

The electrode materials of lithium-ion batteries (LIBs) have been widely studied and applied owing to their higher energy densities [22–28]. Among all the candidates, the lithium manganese oxide LiMn_2_O_4_ (LMO) has the great benefit of a stable spinel structure with 3-dimensional ion diffusion channels, low expense, environmental friendliness, easy accessibility, and large specific capacity, making it a promising candidate as a capacitive deionization electrode [29, 30]. Unfortunately, its cycling stability is still unsatisfactory due to the severe dissolution of Mn upon the discharge/charge process [31, 32]. Currently, oxide layer coating and cationic substitution have been employed to suppress the dissolution of Mn [33, 34]. However, these methods suffer from nonuniform coating layers and complex preparation procedures, respectively [35, 36]. Interestingly, the facet control has been demonstrated to be an effective way to suppress the dissolution of Mn because the manganese ions are more densely arranged in the lattice plane. Although epitaxial growth, hydrothermal-calcination method, and chemical-template method have been used to achieve facet control, the synthesized routine is still complex and difficult to incorporate into industrial applications [37–39].

Herein, we present a facile in situ carbon-incorporated ball-milling method of preparing a LiMn_2_O_4_ material with highly exposed (111) facets. Density functional theory (DFT) calculations have demonstrated that the (111) facet possesses the highest Gibbs free energy change of Mn dissolution and the lowest surface energy among the major facets of the LiMn_2_O_4_ crystal, thus implying that (111) could be the most stable facet. The obtained LiMn_2_O_4_/C composites exhibited highly exposed (111) facets with higher conductivity and significantly improved cycling ability. LiMn_2_O_4_/C retained a stable capacity during 200 cycles in 10 mM LiCl aqueous solution, while bulk LiMn_2_O_4_ only maintain 42.29% of the initial capacity by then, and the Mn dissolution of LiMn_2_O_4_/C was 81.37% lower during the cycling test. With the assistance of an ion-exchange membrane, we constructed a desalination system based on the lithium extraction/insertion reactions of the LiMn_2_O_4_/C cathode and achieved an ultrahigh salt removal capacity of 117.3 mg g^−1^ compared to other reported battery capacitive deionization electrodes.

## Experimental and Calculation

### Materials

NaCl (99.5%), LiCl (≥ 99%), N-Methylpyrrolidone (NMP, analytical grade), and silicon powder were purchased from Aladdin Corp. Spinel lithium manganese oxide (LMO) was purchased from Shanghai Ziyi Co., Ltd. Activated carbon (XFP01) was purchased from XFNANO Co., Ltd. Carbon black was purchased from TIMCAL Graphite & Carbon, and polyvinylidene fluoride (PVDF, HSV900) was purchased from MTI Corp. Anion-exchange membrane (AMX) was purchased from ASTOM Corp. Carbon paper (H-060) was purchased from TORAY Group.

### Synthesis of LiMn_2_O_4_/C

1.0 g LiMn_2_O_4_ and 0.5 g carbon black were mixed and then milled for 6 h in a planetary ball mill (YXQM-4L, MITR) under room temperature.

### Fabrication of Electrodes

Active material, carbon black, and PVDF were ground in an agate mortar for 15 min with a mass ratio of 8:1:1; then, NMP was added to the mixture and ground to form a homogeneous slurry, which was then painted onto the current collector (carbon paper for the electrochemical test or the titanium plate for the desalination test). The current collectors were kept at 90 °C for 4 h to remove the solvent.

### Electrochemical Test

All the electrochemical tests were carried out using an electrochemical workstation (Multi Autolab/M204, Metrohm). The frequency range of the impedance spectroscopy (EIS) test was set to be 10^5^–10^–2^ Hz. The diffusion coefficient of lithium ion is calculated with the following equation:

1 $$D = \left( {R^{2} \times T^{2} } \right)/\left( {2A^{2} \times n^{4} \times F^{4} \times C^{2} \times \sigma^{2} } \right)$$

in which *D* (cm^2^ s^−1^) refers to the diffusion coefficient of the ion, *R* is the gas constant (8.314 J mol^−1^ K^−1^), *T* (K) represents the absolute temperature, *A* (1 cm^2^) denotes the area of the working electrode, *n* is the number of the transferred electrons when one molecule is oxidated or reduced, *F* stands for the Faraday constant (96,485 C mol^−1^), *C* (0.0236 mol cm^−3^) refers to the concentration of Li^+^ in solid, and *σ* (Ω s^−0.5^) is the Warburg factor [40].

### Deionization Test

The total mass of the active materials in the cathode and anode was controlled to be 100 mg with a mass ratio of 1:2. The applied potential was added by the electrochemical workstation. The measurements of concentration changes of chloride and lithium ions were taken with ion chromatography (883 Basic IC Plus, Metrohm). The salt adsorption capacity (*SAC*, mg g^−1^) could be defined as:

2 $${\text{SAC}} = \left( {C_{0} - C_{\text{f}} } \right) \times V/m$$

where *C*_*0*_ and *C*_f_ denote the initial and final concentration of the salt solution (mg L^−1^), *V* (L) is the volume of the solution, and *m* (g) is the total mass of the active materials. The salt adsorption rate (*SAR*, mg g^−1^ min^−1^) can be represented as:

3 $${\text{SAC}} = {\text{SAC}}/T$$

in which *T* (min) is the desalination reaction time and energy consumption *W* (Wh g^−1^) is calculated as follows:

4 $$W = \left( {1000 \times U \times \int {I dt} } \right)/\left( {3600 \times {\text{SAC}} \times m} \right)$$

where *U* (V) refers to the charged voltage, *I* (A) is the current during the deionization experiment, *t* (s) denotes the reaction time. The charge efficiency of the deionization cell *Λ* is calculated by:

5 $$\Lambda = \left( {{\text{SAC}} \times m \times F} \right)/\left( {1000 \times M_{\text{NaCl}} \times \int {I dt} } \right)$$

in which *M*_NaCl_ is the molar mass of NaCl (g mol^−1^).

### Characterization

The morphologies and structures of the materials were observed under field emission scanning electron microscope (FESEM, JSM-7900F, JEOL) and transmission electron microscope (TEM, Talos F200X, FEI). Brunauer–Emmett–Teller (BET) results were obtained with surface area and pore size analyzer (KUBOX1000, Bjbuilder). The carbon content of the material was tested with infrared carbon and sulfur analyzer (CS844, LECO). The electrical conductivities of the materials were measured using a four-probe electrical resistivity tester (ST2772-SZ, Suzhou Jingge). Crystal structure information was acquired with X-ray diffraction tests (XRD, Empyrean 2, PANalytical). The Mn valence information was obtained with X-ray photoelectron spectroscopy tests (XPS, ESCALAB 250Xi, Thermal Fisher Scientific). 

### First-Principles Calculations

All structural optimization and total energy calculations were performed using the Vienna ab initio simulation package (VASP) based on density functional theory (DFT) [41]. The electron–ion interaction was treated by the projector-augmented wave (PAW) method, and the plane-wave basis was set to a cutoff energy of 500 eV [42]. The Perdew–Burke–Ernzerhof (PBE) exchange–correlation functional was employed for the generalized gradient approximation (GGA) [43]. A vacuum slab of 15 Å was applied along the *z* direction to allow the interaction between repeated slabs to be ignored. The reciprocal space *k*-point mesh interval was approximately 0.04 Å^−1^. The force and energy convergence criterion were set to 0.02 eV Å^−1^ and 10^−5^ eV, respectively. To treat localized 3*d* orbitals, we applied Hubbard *U* corrections with *U*_eff_ = 5 eV for Mn atoms [44–46]. In addition, the schematic images of the crystal structures were drawn with the software Visualization for Electronic and Structural Analysis (VESTA) [47].

The stoichiometric slabs with symmetrically equivalent surfaces of (111), (311), (400), and (440) facets obtained from bulk LiMn_2_O_4_ were fully relaxed, and the surface energy *γ* is described as:

6 $$\gamma = \left( {E_{{{\text{slab}}}} - E_{{{\text{bulk}}}} } \right)/2A$$

where *E*_slab_ and *E*_bulk_ are the total energies of slab and bulk with the same atomic number, respectively, and $\it A$ is the surface area. The Gibbs free energy $\Delta\it G$ for the formation of a Mn atomic vacancy at the surface is calculated as:

7 $$\Delta G = E\left( {{\text{Li}}_{x} {\text{Mn}}_{2x - 1} {\text{O}}_{4x} } \right) + E\left( {{\text{Mn}}} \right) - E\left( {{\text{Li}}_{x} {\text{Mn}}_{2x} {\text{O}}_{4x} } \right)$$

where *E(*Li_*x*_Mn_2*x*_O_4*x*_*)*, *E(*Li_*x*_Mn_2*x-1*_O_4*x*_*)*, and *E(*Mn*)* are the total energies of intact surface, surface with Mn atomic vacancy, and Mn atom, respectively.

## Results and Discussion

### Preparation of LiMn_2_O_4_/C with Exposed (111) Facets

**Fig. 1:**
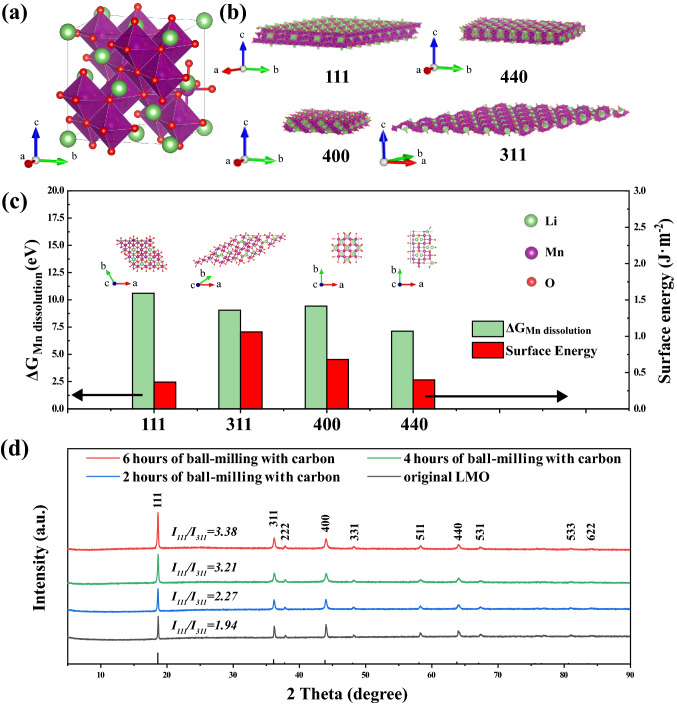
**a** Crystal structure of LiMn_2_O_4,_ purple for manganese ions, green for lithium ions, and red for oxygen ions. **b** (111), (311), (400), and (440) facets. **c** Calculated results of the Gibbs free energy changes of Mn dissolution reactions and the surface energies of different lattice planes. **d** Comparison of XRD patterns of LMO after the ball-milling processes of different time lengths with the incorporation of carbon

As schemed in Fig. 1a, the manganese ion just locates in the octahedral structure consists of oxygen ions. The Gibbs free energy changes (Δ*G*) of Mn dissolution and the surface energies of predominant lattice planes were determined via DFT using VASP [48, 49]. According to the calculated result, the (111) facet has the highest Δ*G* (10.60 eV) and the lowest surface energy (0.37 J m^−2^) among the representative facets (Fig. 1c), implying that the (111) facet could be the most stable lattice plane. As the ball-milling time was increased, the intensity ratio of (111) compared to other peaks increased (Fig. 1d, Table S1), which was in good agreement with the calculated results. The same amount of material was used in each XRD sample, and the intensities of all the peaks of the ball-milled LiMn_2_O_4_ particles without carbon incorporation decreased (Fig. S1). The intensity of the (111) peak of LiMn_2_O_4_/C increased in comparison with that in the pristine LMO XRD pattern as shown in Fig. 1d, from which the protection of crystals by carbon could be inferred [50–52]. LiMn_2_O_4_/C composites with highly exposed (111) facets were successfully obtained after 6 h of ball-milling.

### Structural and Cyclic Stabilities of LiMn_2_O_4_/C

**Fig. 2:**
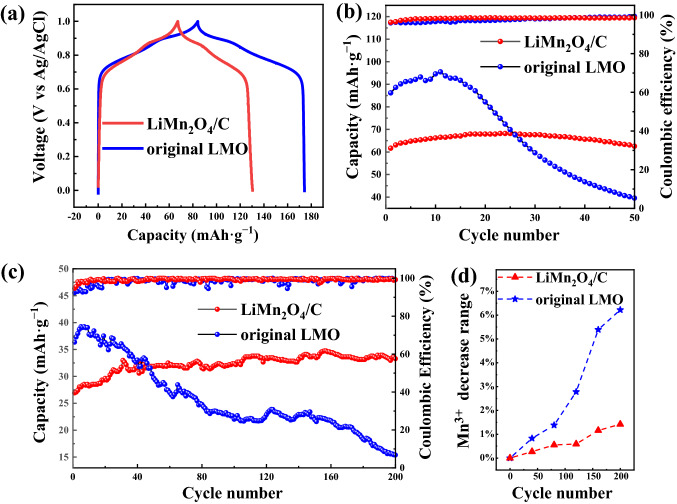
**a** Galvanostatic charge–discharge (GCD) profiles of LiMn_2_O_4_/C and bulk LMO in 1.0 M LiCl solution. Cycling performances of the two materials in **b** 1.0 M and **c** 10 mM LiCl solutions. **d** Mn^3+^ content decreases during the cycling tests in 10 mM LiCl solution

All electrochemical tests were conducted in a three-electrode system with an Ag/AgCl electrode as the reference electrode and LiCl aqueous solution as the electrolyte. As shown in Fig. 2a, the charge capacity of LiMn_2_O_4_/C was 67.5 mAh g^−1^, almost 20% smaller than that of bulk LMO. This finding was in accordance with the infrared carbon and sulfur analyzer result that the carbon mass content of LiMn_2_O_4_/C was 33.29%, and similar voltage plateaus were observed for the two materials. Cycling tests (current density: 100 mA g^−1^) were carried out to examine the electrochemical stabilities of the materials. Figure 2b shows that the bulk LMO retained only 45.86% of the initial capacity in the 50th cycle in the 1.0 M LiCl solution, while little decay was observed for LiMn_2_O_4_/C. To confirm the cycling abilities of the two cathodes at the concentration of the deionization experiment, galvanostatic charge–discharge (GCD) tests in the 10 mM LiCl solution were performed. As shown in Fig. 2c, the initial capacities of the materials were lower than those in 1.0 M electrolyte because of the lower solution conductivity. LiMn_2_O_4_ retained 42.29% of the initial capacity after 200 cycles, while in contrast, the capacity of LiMn_2_O_4_/C remained steady throughout cycling, thus indicating robust electrochemical stability.

In the cycling test in 10 mM LiCl solution, LiMn_2_O_4_/C had a suppressed Mn dissolution of 0.058 mg per gram of material over the cycles tested by inductively coupled plasma mass spectrometry (ICP-MS), which was 81.37% lower than that of bulk LMO, suggesting a more stable structure. The variation in the Mn valence during the cycling tests was measured by XPS (Figs. 2d and S2, S3). The content of unstable Mn^3+^ ions decreased slower in LiMn_2_O_4_/C, a sign of alleviated Mn dissolution. A tiny increase in the specific capacity of LiMn_2_O_4_/C appeared in the GCD test, which was owing to the activation of lithium ions. The changes in the peak shifts of XRD patterns after charging in different cycles of the LiMn_2_O_4_/C material were smaller compared to those of bulk LMO (Figs. S4 and S5), revealing a steadier structural transformation with a more constant amount of lithium ions in the deintercalation/intercalation process. Taken together, the LiMn_2_O_4_/C material possessed higher structural and electrochemical stabilities.

The morphologies of the original spinel LMO and LiMn_2_O_4_/C are shown in Fig. S8. The LMO particle in LiMn_2_O_4_/C had a size of approximately 10–15% of that of bulk LMO, and the specific surface area of the LiMn_2_O_4_/C material was 26.46 m^2^ g^−1^ by the BET method, nearly 34.5 times of that of LiMn_2_O_4_. Under TEM observation, the obvious spinel LiMn_2_O_4_ structures were surrounded by the appropriate carbon network, which could not only facilitate charge transfer by tight connection with crystals (Fig. S9) but also reserve necessary spaces for ion transfer and buffering of the expansion and contraction of LMO during reactions and collisions in the ball-milling (Fig. S9a). The interplanar distance of the exposed lattice planes of LiMn_2_O_4_/C was 0.48 nm, demonstrating the stable (111) facets.

**Fig. 3:**
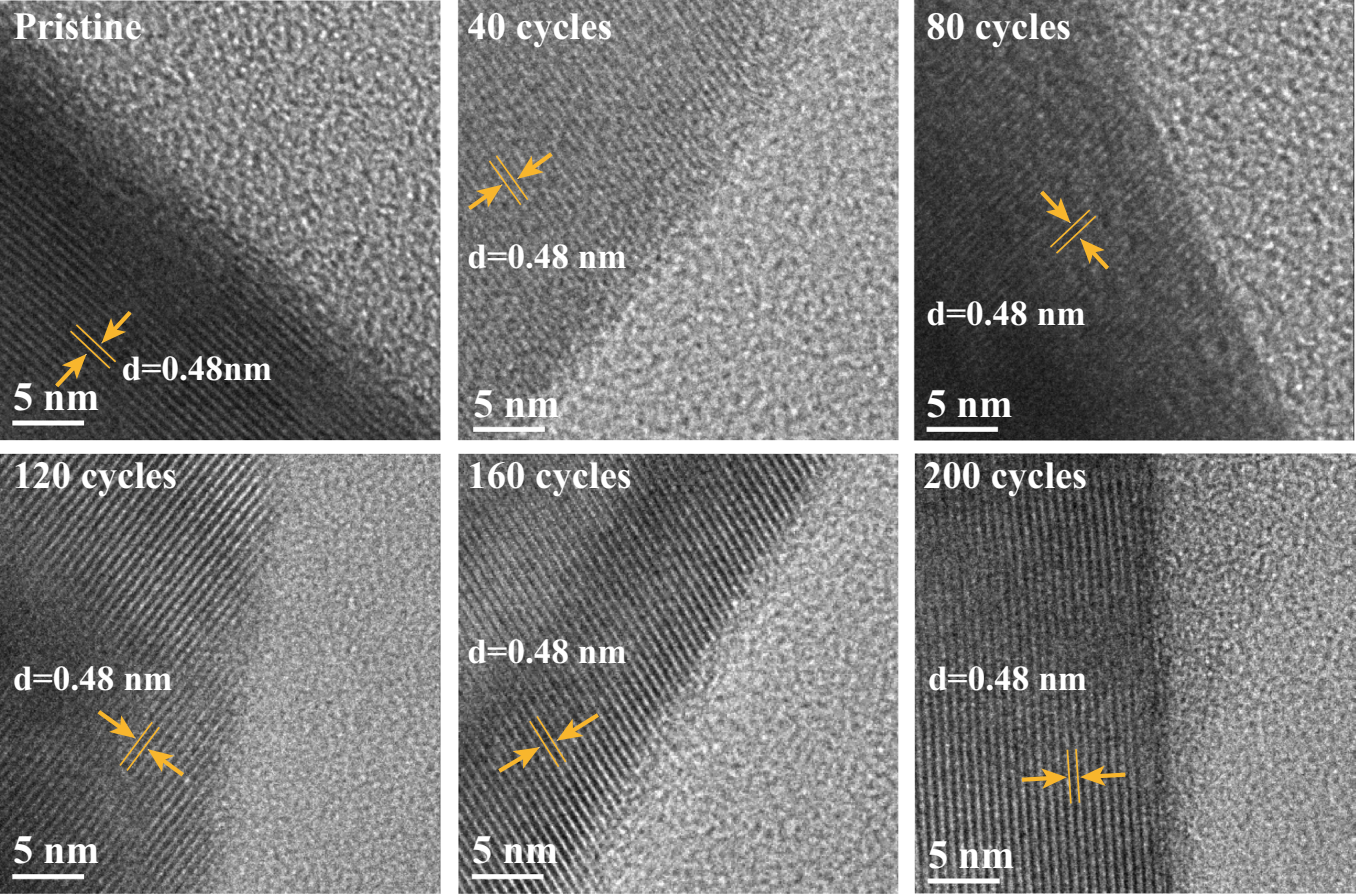
TEM images of crystal surfaces of LiMn_2_O_4_/C after different cycles in 10 mM LiCl solution

As shown in Figs. S10 and S11, the crystal structures of LMO collapsed during cycling while those of LiMn_2_O_4_/C remained stable under scanning electron microscope (SEM) observation. For further insight, the two materials were observed using TEM at different cycles. The crystal surface of the original LMO consisted of a variety of lattice planes (Fig. S12) and suffered drastic degradation as the charge–discharge reactions continued (Fig. S13). In contrast, as the (111) facet became the dominant lattice plane in LiMn_2_O_4_/C, it was exposed just at the surface of the crystals, protecting the material frameworks (Fig. 3). The unique structure of LiMn_2_O_4_/C determined the nature of the promoted stability.

8 $${\text{LiMn}}_{2} {\text{O}}_{4} \rightleftharpoons {\text{Li}}_{1 - x} {\text{Mn}}_{2} {\text{O}}_{4} + x{\text{Li}}^{ + } + xe^{ - }$$

The redox behaviors of the two materials are represented above. The cyclic voltammetry curves of spinel LMO and LiMn_2_O_4_/C (Fig. S14) with the upper voltage scan range limited to 1.0 V to prevent severe water splitting both exhibit two pairs of redox peaks, signifying the insertion/extraction processes of lithium ions at the 16c and 8a sites. Nevertheless, in comparison with the original LMO, the redox peaks of LiMn_2_O_4_/C were sharper and the voltage gap between the cathode peak and the anode peak in each pair was smaller, indicating better reversibility in electrochemical reactions [53]. As shown in the EIS result (Fig. S15), the charge transfer resistance of the LiMn_2_O_4_/C electrode was obviously reduced. With the incorporation of carbon, the electrical conductivity of the material rose from 3.85 × 10^–3^ to 500 S m^−1^ based on the powder resistivity tester result. Yet it was not completely revealed in the EIS profile, likely due to the conductive agent that improved the conductivity already during the fabrication of both electrodes. The Warburg impedance was determined from the Nyquist plots in the low-frequency region, with which the diffusion coefficients of lithium-ion (D-Li^+^) influenced by concentration polarization can be calculated by Eq. (1) [54]. Figure S16 shows the linear fitting of *Z*’ and the reciprocal square root of angular frequency (*ω*^−1/2^), the slope of which refers to the relevant Warburg factor *σ* [55]. The Warburg factor values of LiMn_2_O_4_/C and the original LMO were calculated to be 126.26 and 71.64 Ω s^−0.5^, and the D-Li^+^ values were 4.62 × 10^–15^ and 1.24 × 10^–14^ cm^2^ s^−1^, respectively. The D-Li^+^ of the original material was slightly larger, which might be due to diffusion blockage resulting from the added carbon on the solid–liquid interface between the crystal and the electrolyte. This observation further suggests that the decrease in the electrochemical polarization was a major mechanism of the enhancement of the electrochemical reversibility of the material. Apart from the lower charge transfer resistance, the structural stability of LiMn_2_O_4_/C with less Mn dissolution could provide more stable active sites and pathways for lithium ions. This might also explain why the reaction was more reversible when the ion diffusion coefficient was lower. Conversely, enhanced reaction reversibility is important for structural stability as well by allowing the same quantity of lithium ions to participate in the reaction no matter whether the charging or discharging is occurring. The mutual support of the structural stability and reaction reversibility could back up the electrochemical stability, with which we could struggle for a suitable path to utilize LIB materials for long-term use in electrochemical deionization desalination.

### Desalination Performance

**Fig. 4:**
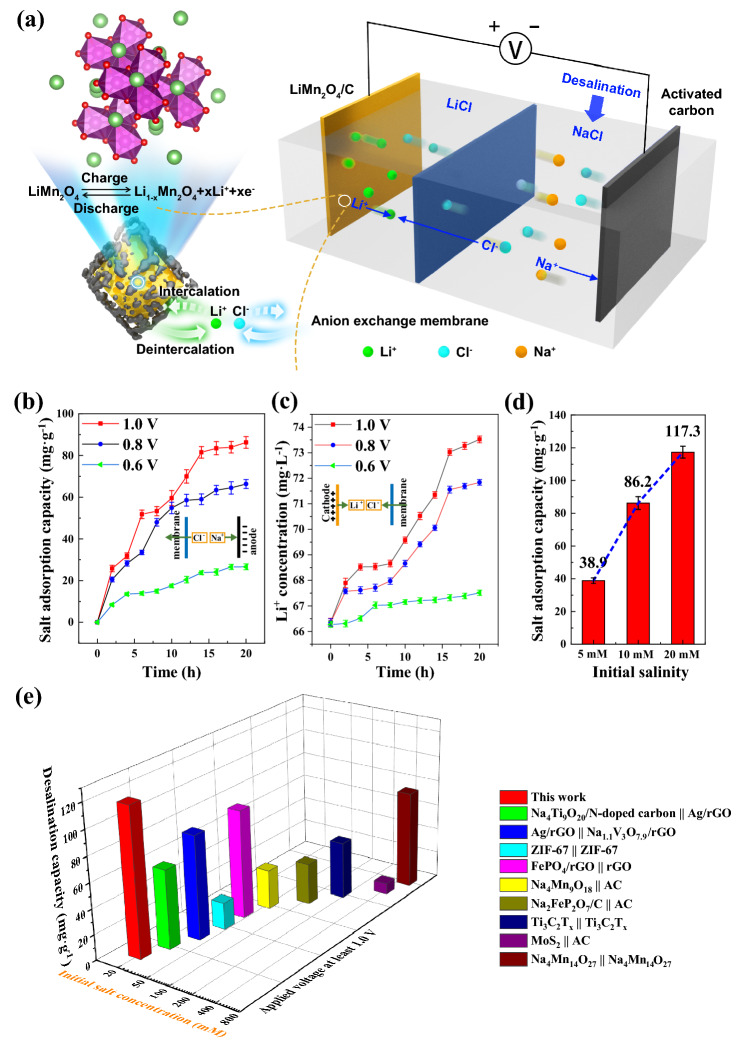
**a** Schematic diagram of the deionization cell. **b** Salt removal capacities of the deionization cell and **c** lithium release of LiMn_2_O_4_/C in 10 mM LiCl solution during desalination at different voltages. **d** Salt removal performances with a variation of initial NaCl concentration at 1.0 V. **e** Desalination performance comparison of different battery electrodes for deionization

The deionization cell was divided by the anion-exchange membrane into two compartments as presented in Fig. 4a, one side with 100 ml LiCl aqueous solution and the other side with 125 mL 10 mM NaCl aqueous solution. The LiMn_2_O_4_/C cathode (5 × 5 cm^2^) was immersed in LiCl solution with activated carbon (AC) in NaCl solution as the anode (5 × 5 cm^2^). When a voltage was applied, the lithium ions were released into the catholyte, attracting the chloride ions from the other side to pass through the membrane into the LiCl solution, and the sodium ions blocked by the membrane in NaCl solution were adsorbed by the AC electrode at the same time; thus, the NaCl solution was desalinated.

The desalination performances of the deionization system with the LiMn_2_O_4_/C electrode at 1.0, 0.8, and 0.6 V with an initial NaCl concentration of 10 mM over the whole reaction processes are shown in Fig. 4b. The desalination capacities increased throughout the deionization processes. A desalination capacity of 86.2 mg g^−1^ was obtained at a voltage of 1.0 V after 20 h of charging; the capacity remained almost unchanged compared to that of the system with bulk LMO (91.0 mg g^−1^), although nearly one-third of the LiMn_2_O_4_/C mass was carbon. As the electric voltage applied to the cathode was increased, the salt removal performance was enhanced, and the electric field and the strengthened lithium release both drew the oppositely charged ions (Fig. 4c). The salt removal capacity at 0.6 V (26.6 mg g^−1^) was much lower than those at 1.0 and 0.8 V (66.3 mg g^−1^), because only a few lithium ions were released from the cathode as 0.6 V just occupied the lower edge of the electrochemical plateau of the cathode material. The higher the applied voltage was, the higher the salt removal rate became, and the desalination rates slowed as the deionization experiments were continued (Fig. S17); this observation was consistent with the salt adsorption capacity plateaus in the later hours, as illustrated in Fig. 4b. As shown in Fig. 4d, the deionization performance was positively correlated with the salt content of the NaCl solution, which accelerated the charge and ion transfer in the solution with higher electrical conductivity. Ultimately, a high deionization capacity of 117.3 mg g^−1^ in 20 mM NaCl solution at 1.0 V was achieved compared to many other reported desalination performances (Fig. 4e, Table S2) [11, 16, 19, 56–61].

Figure S18 depicts the current variations during the desalination processes with different cathodes, from which the charge efficiency and energy consumption were calculated. As described in Fig. S20, the charge efficiency of the system with LiMn_2_O_4_/C during desalination was 80.46%, much higher than that of the system with original LMO (54.92%), following the enhanced charge transfer. The energy consumption of the cell with LiMn_2_O_4_/C was more stable throughout the desalination process, and the value over 20 h was 0.54 Wh per gram of salt removal. This value was approximately 32.49% lower than that of the cell with the LMO cathode (Fig. S19), indicating a more energy-efficient capacitive deionization electrode with a higher deionization performance.

## Conclusions

In summary, a LiMn_2_O_4_/C electrode material was employed as the cathode in the electrochemical deionization system for desalination, and it delivered a high salt removal performance of 117.3 mg g^−1^. The electrode maintained a high stability for approximately 200 cycles in the 10 mM LiCl aqueous solution, while the capacity dropped quickly after the initial 20 cycles for the pristine LMO. The superior electrochemical stability is attributed to the improved conductivity and the low dissolution of Mn, which is evidenced by the DFT calculations. This one-step method of preparing LMO with highly exposed (111) facets at a low cost might open a new avenue for desalination.

## Supplementary Information

Below is the link to the electronic supplementary material.

Supplementary file1 (PDF 2321 kb)
