# Supplementary material for: Facet-Controlled LiMn2O4/C as Deionization Electrode with Enhanced Stability and High Desalination Performance
Source: Nanomicro Lett. 2022 Aug 23;14:176. doi: 10.1007/s40820-022-00897-3 (PMC9399334; doi:10.1007/s40820-022-00897-3)
Supplement: Supplementary file 1 — Supplementary file1 (PDF 2321 kb) [file 40820_2022_897_MOESM1_ESM.pdf]

Supporting Information for

Facet-Controlled $LiMn_2O_4$/C as Deionization Electrode with Enhanced Stability and High Desalination Performance

Yuxin Jiang[1], Liyuan Chai[1, 2, 3], Dehe Zhang[4], Fangping Ouyang[4, 5], Xiangyuan Zhou[1], Sikpaam I. Alhassan[1], Sailin Liu[6], Yingjie He[1], Lvji Yan[1], Haiying Wang[1, 2, 3, *], and Wenchao Zhang[1, 2, 3, *]

[1]School of Metallurgy and Environment, Central South University, Changsha 410083, P. R. China

[2]Chinese National Engineering Research Center for Control and Treatment of Heavy Metal Pollution, Changsha 410083, P. R. China

[3]Water Pollution Control Technology Key Lab of Hunan Province, Changsha 410083, P. R. China

[4]School of Physics and Electronics, Hunan Key Laboratory for Super-Microstructure and Ultrafast Process, and Hunan Key Laboratory of Nanophotonics and Devices, Central South University, Changsha 410083, P. R. China

[5]State Key Laboratory of Powder Metallurgy, and Powder Metallurgy Research Institute, Central South University, Changsha 410083, P. R. China

[6]School of Chemical Engineering and Advanced Materials, Faculty of Sciences, Engineering and Technology, The University of Adelaide, Adelaide 5005, Australia

*Corresponding authors. E-mail: haiyw25@csu.edu.cn (Haiying Wang), wenchao.zhang@csu.edu.cn (Wenchao Zhang)

S1 Supplementary Figures and Tables

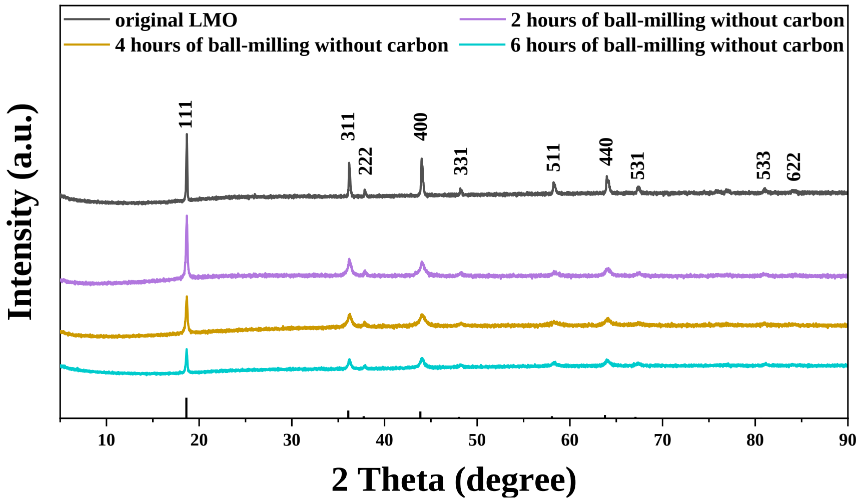

Fig. S1 Comparison of X-ray diffraction (XRD) patterns of LMO after the ball-milling processes of different time lengths without incorporation of carbon

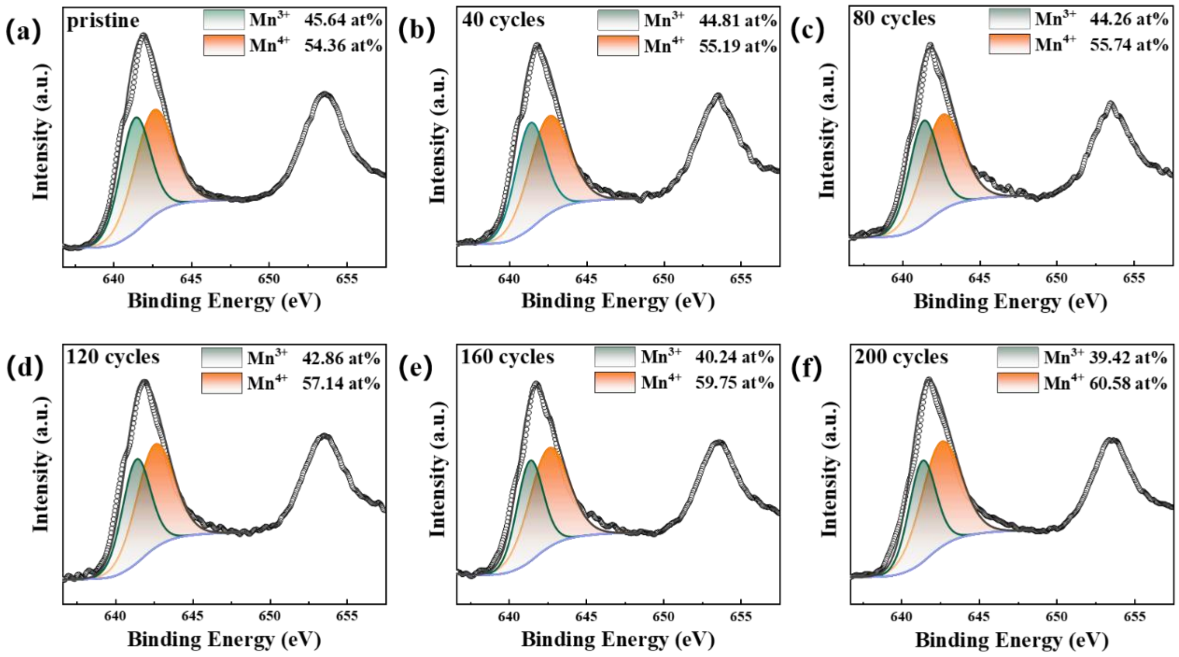

Fig. S2 X-ray photoelectron spectroscopy (XPS) results of Mn2p of original LMO during the cycling tests in 10 mM LiCl solution

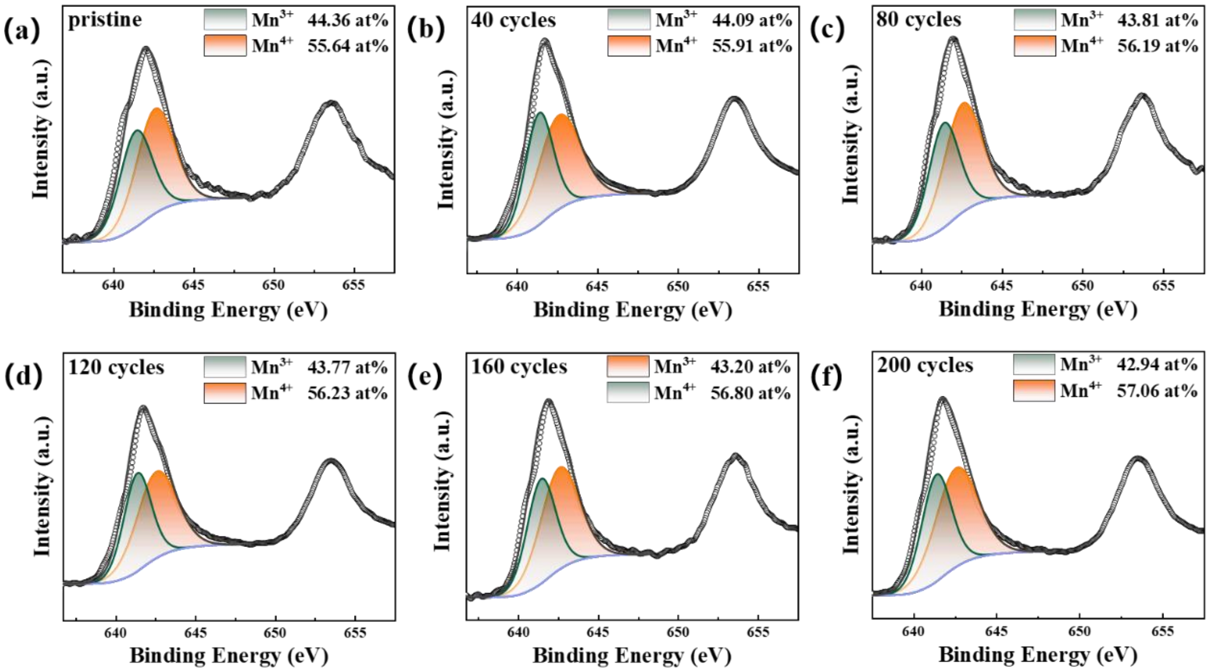

Fig. S3 XPS results of Mn2p of $LiMn_2O_4$/C during the cycling tests in 10 mM LiCl solution

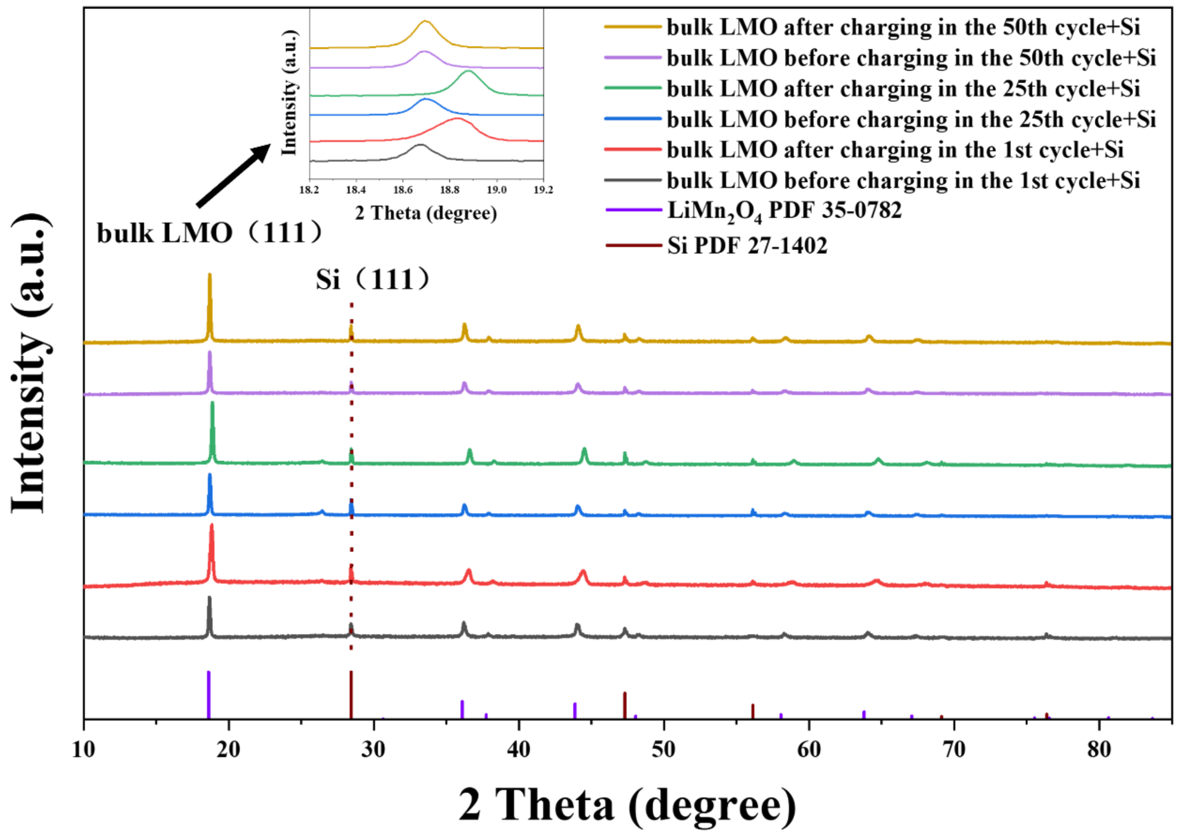

Fig. S4 XRD patterns of the of bulk LMO before and after charging in different cycles (in 1.0 M LiCl solution) mixed with silicon powder

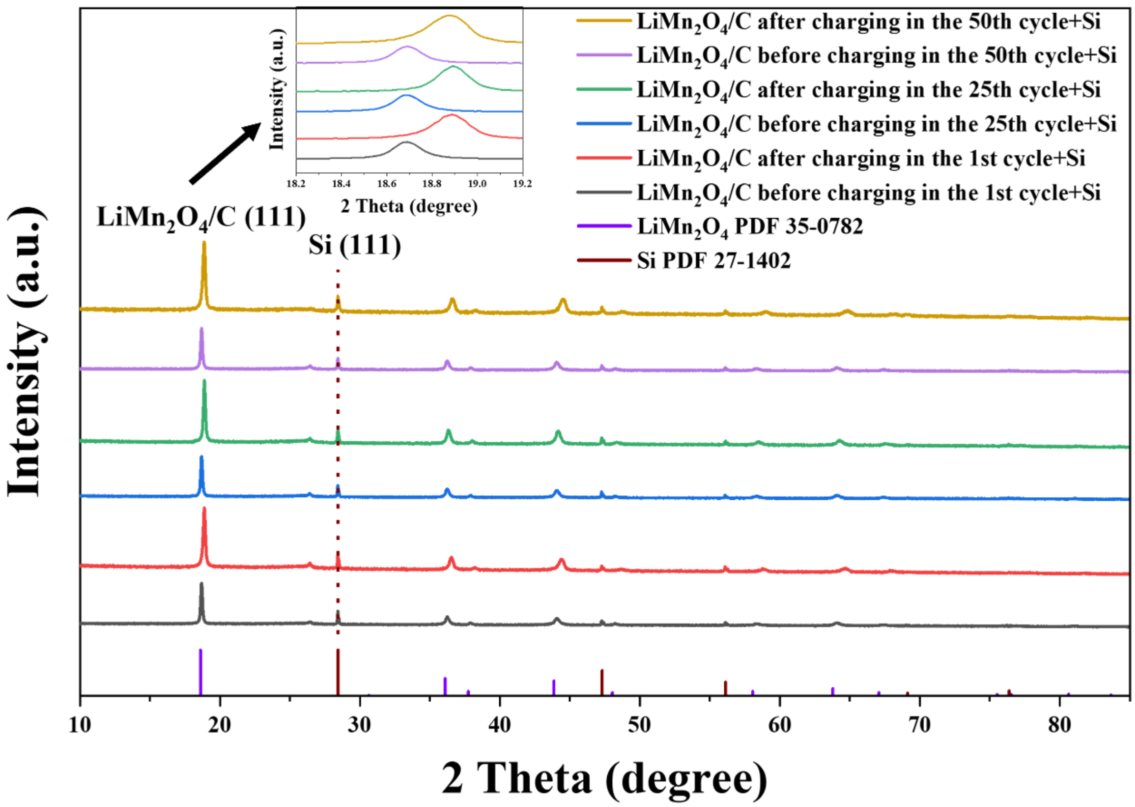

Fig. S5 XRD patterns of $LiMn_2O_4$/C before and after charging in different cycles (in 1.0 M LiCl solution) mixed with silicon powder

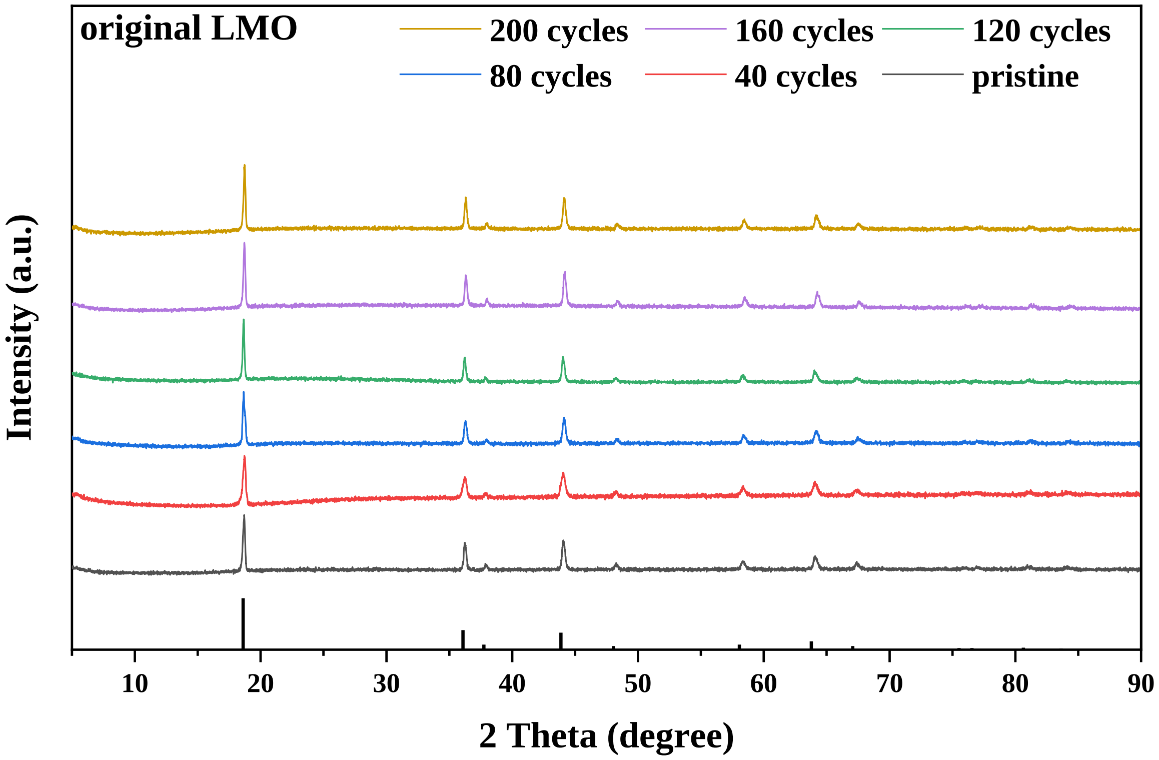

Fig. S6 XRD patterns of LMO during the cycling tests in 10 mM LiCl solution

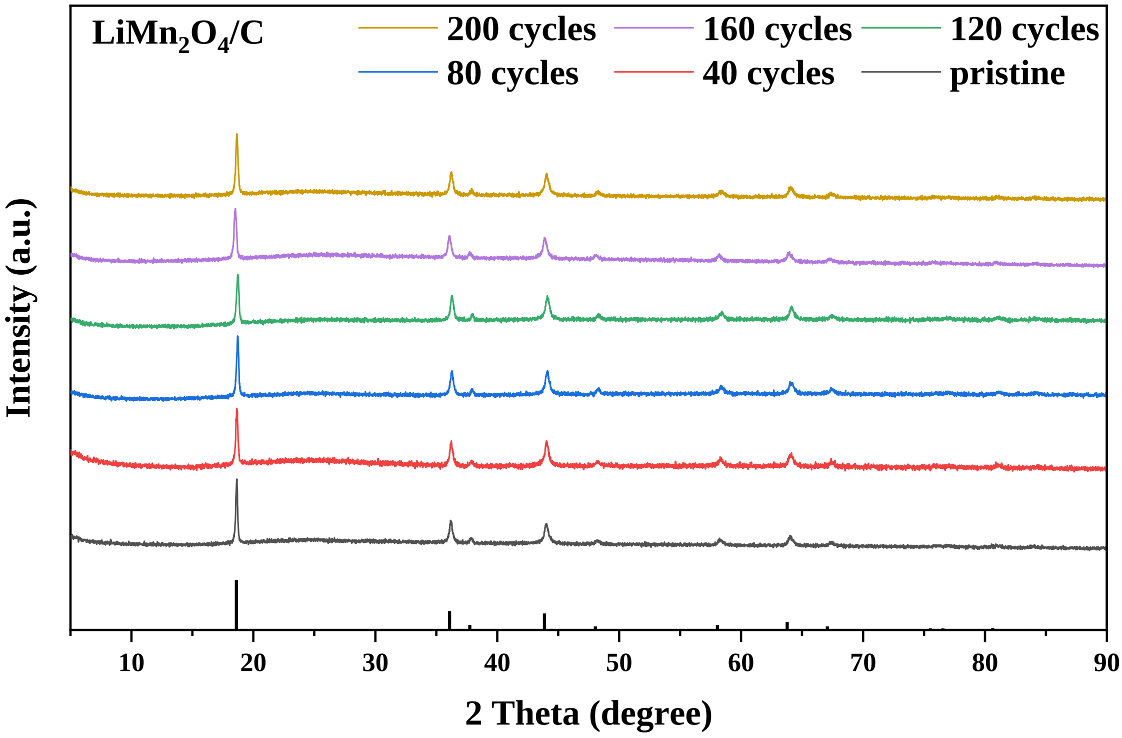

Fig. S7 XRD patterns of $LiMn_2O_4/C$ during the cycling tests in 10 mM LiCl solution

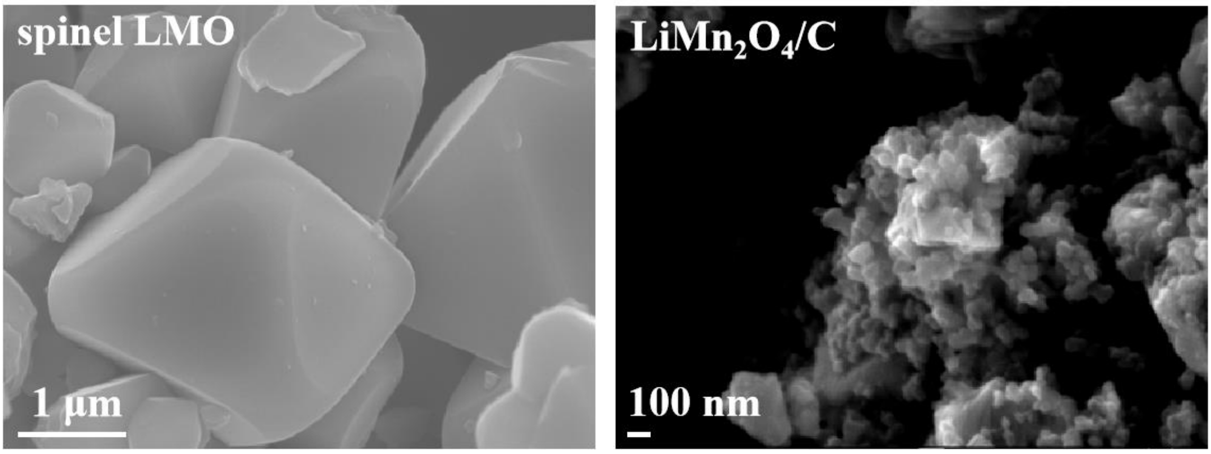

Fig. S8 Scanning electron microscope (SEM) images of LMO and $LiMn_2O_4/C$

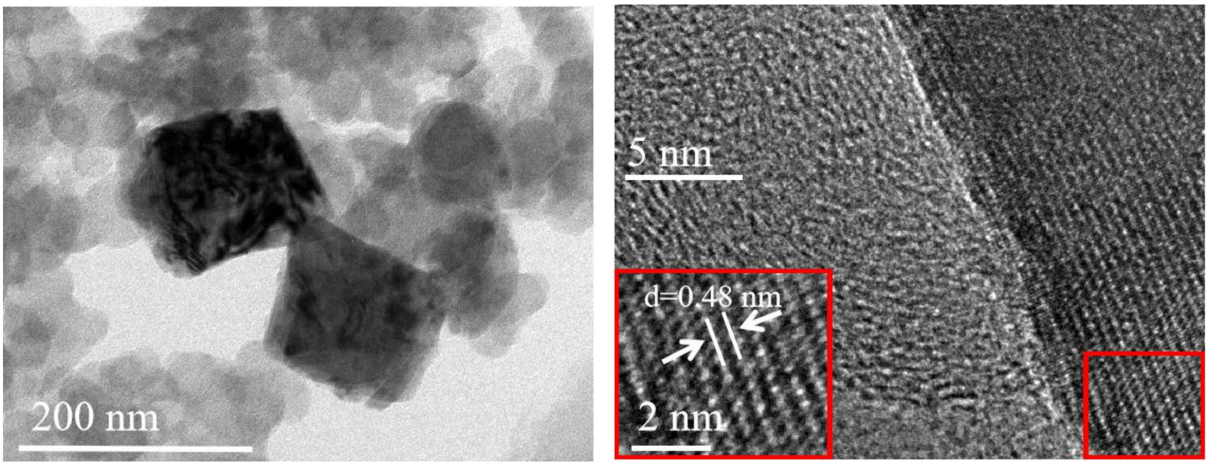

Fig. S9 Transmission electron microscope (TEM) images of $LiMn_2O_4/C$

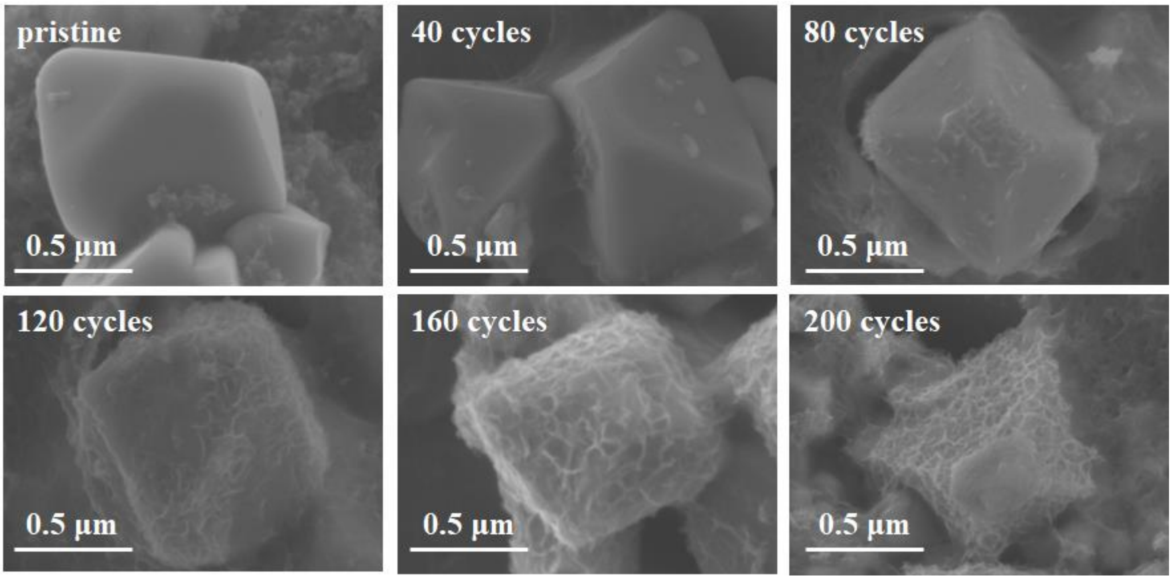

Fig. S10 SEM images of LMO during the cycling tests in 10 mM LiCl solution

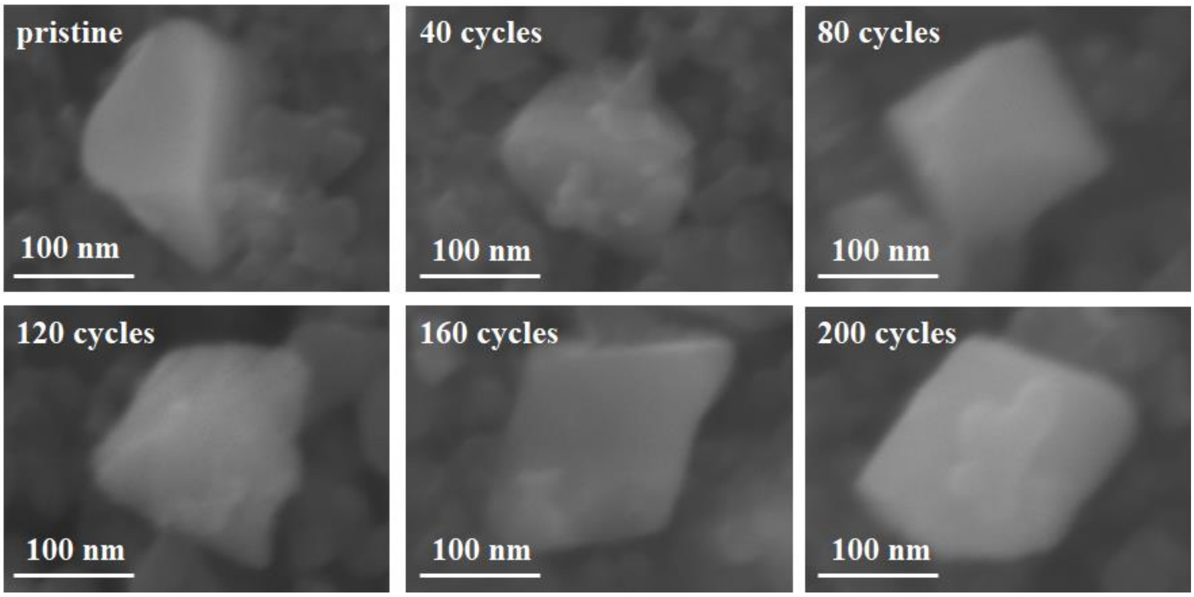

Fig. S11 SEM images of $LiMn_2O_4$/C during the cycling tests in 10 mM LiCl solution

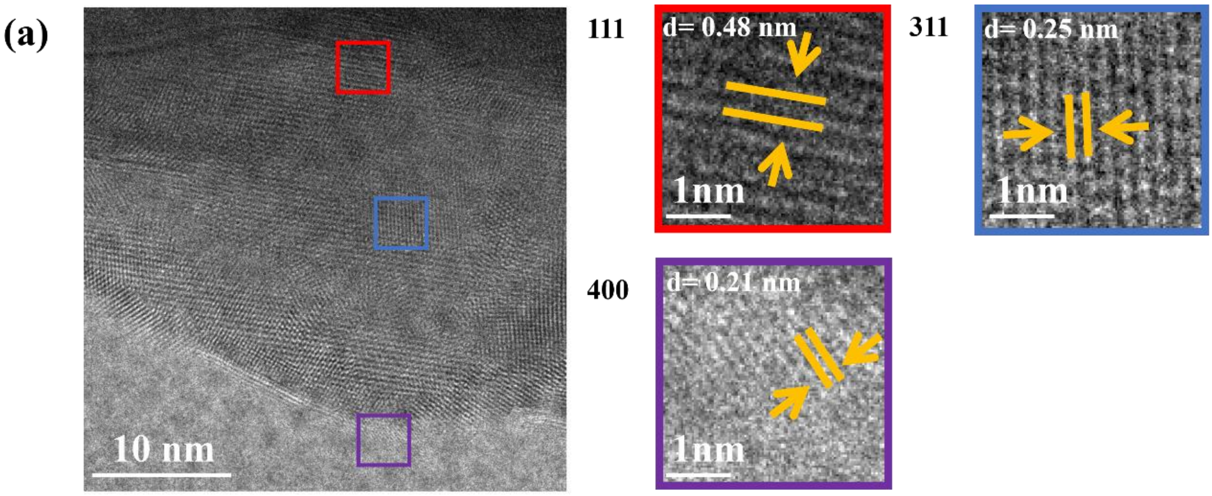

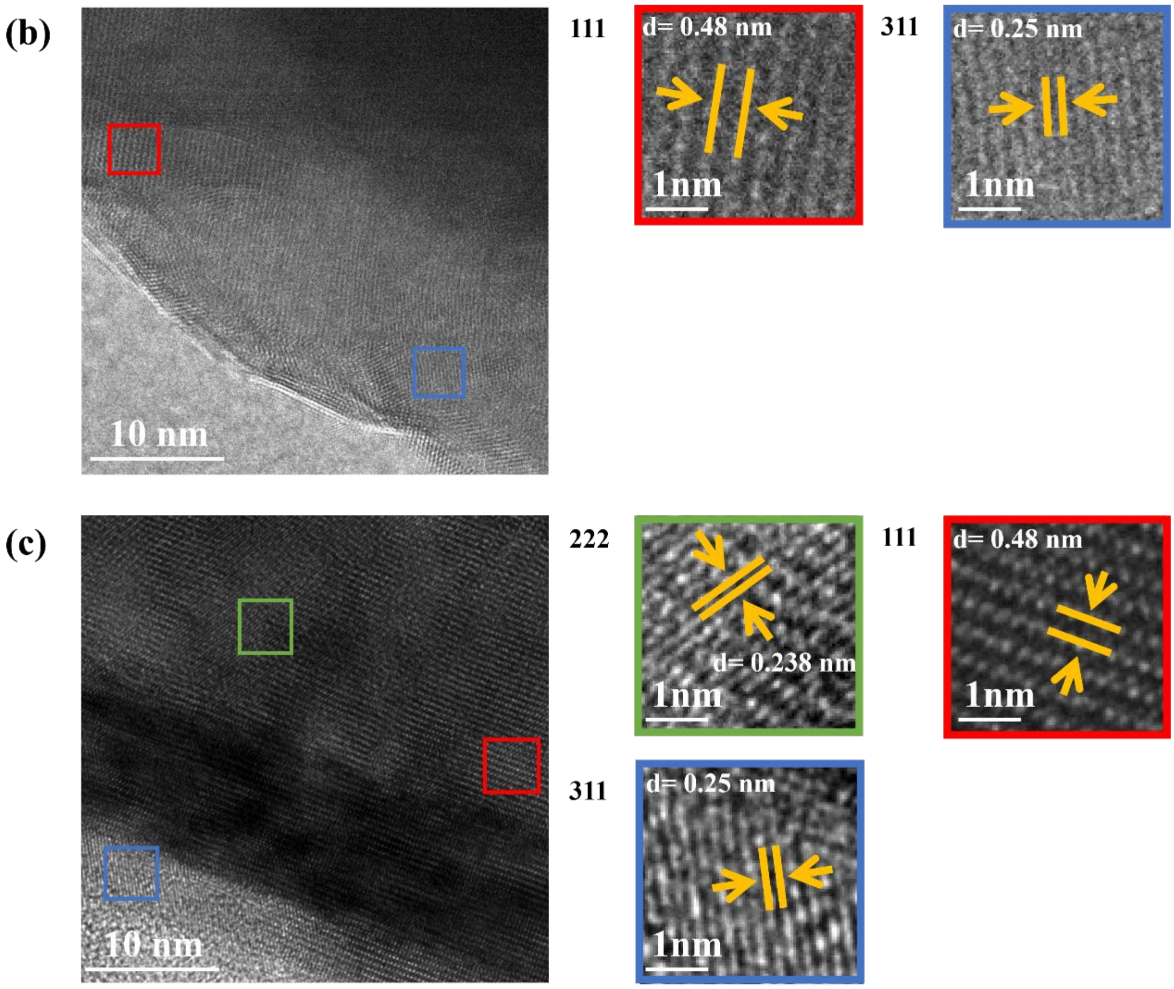

Fig. S12 TEM images of LMO crystal surfaces

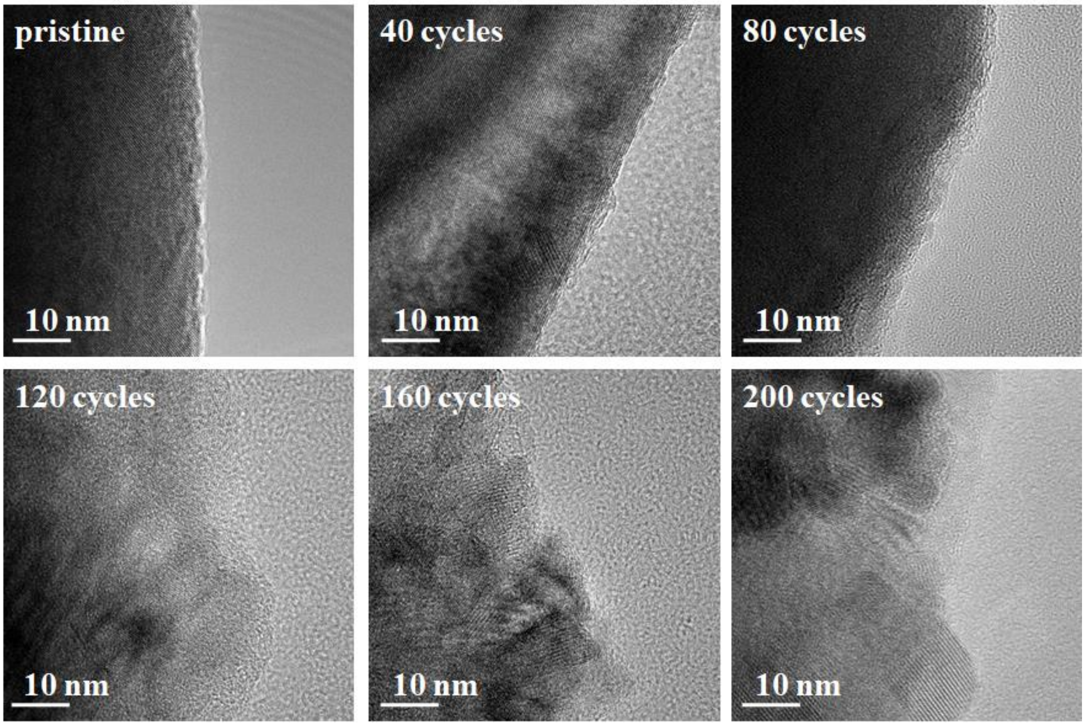

Fig. S13 TEM images of crystal surfaces of LMO after different cycles in 10 mM LiCl solution

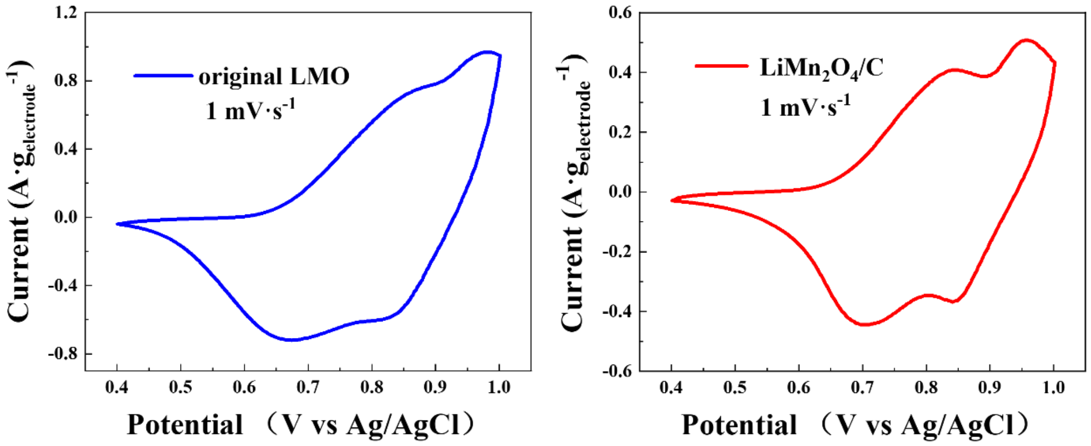

Fig. S14 Cyclic voltammetry results of original LMO and $LiMn_2O_4$/C with a scan rate of 1.0 mV·s^{-1}

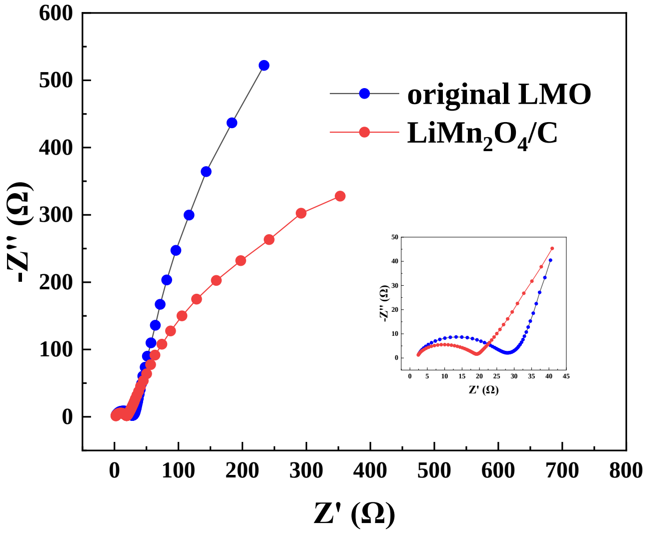

Fig. S15 Comparison of the Nyquist plots

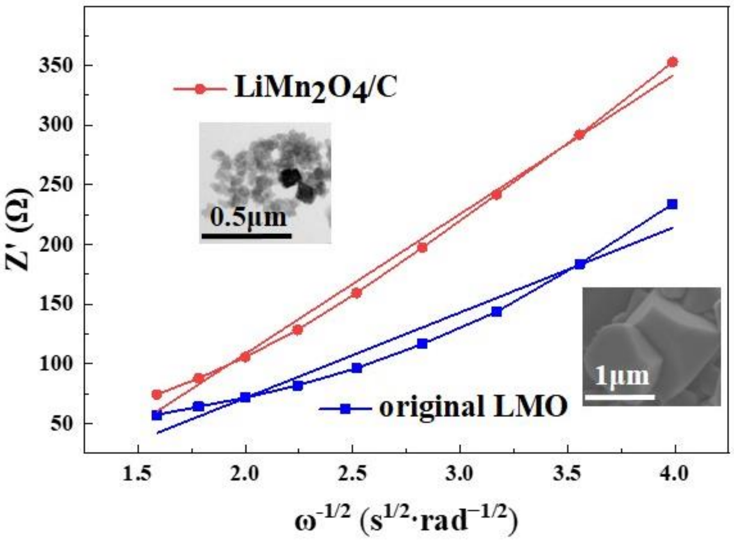

Fig. S16 Fittings of Z' and the reciprocal square root of the angular frequency in the low-frequency region of electrochemical impedance spectroscopy of the materials

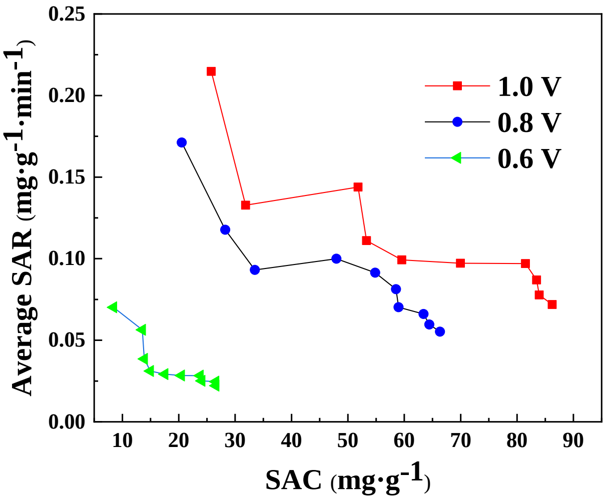

Fig. S17 Deionization Ragone plots

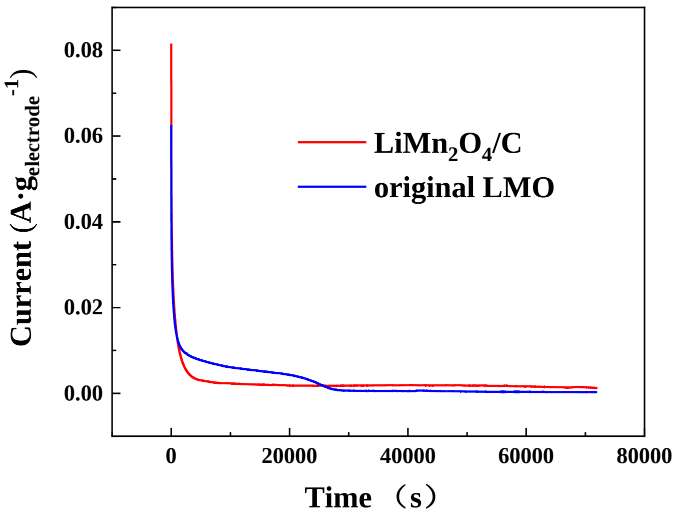

Fig. S18 Constant voltage charging profiles of deionization systems with $LiMn_2O_4$/C or LMO at 1.0 V with an initial salt concentration of 10 mM

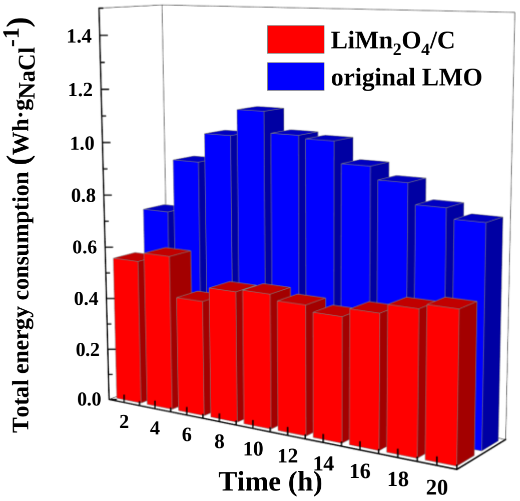

Fig. S19 Energy consumption comparison of the two hybrid deionization systems with different cathodes

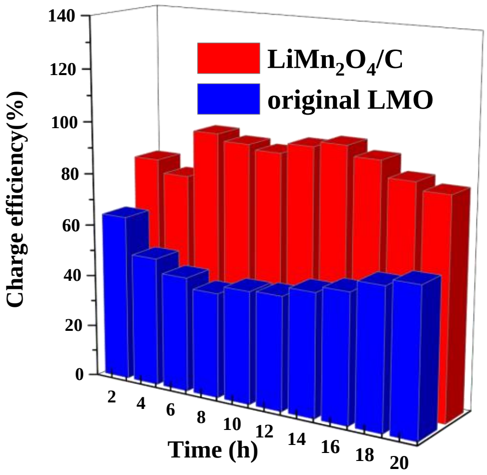

Fig. S20 Charge efficiencies of deionization cells with different cathodes during desalination

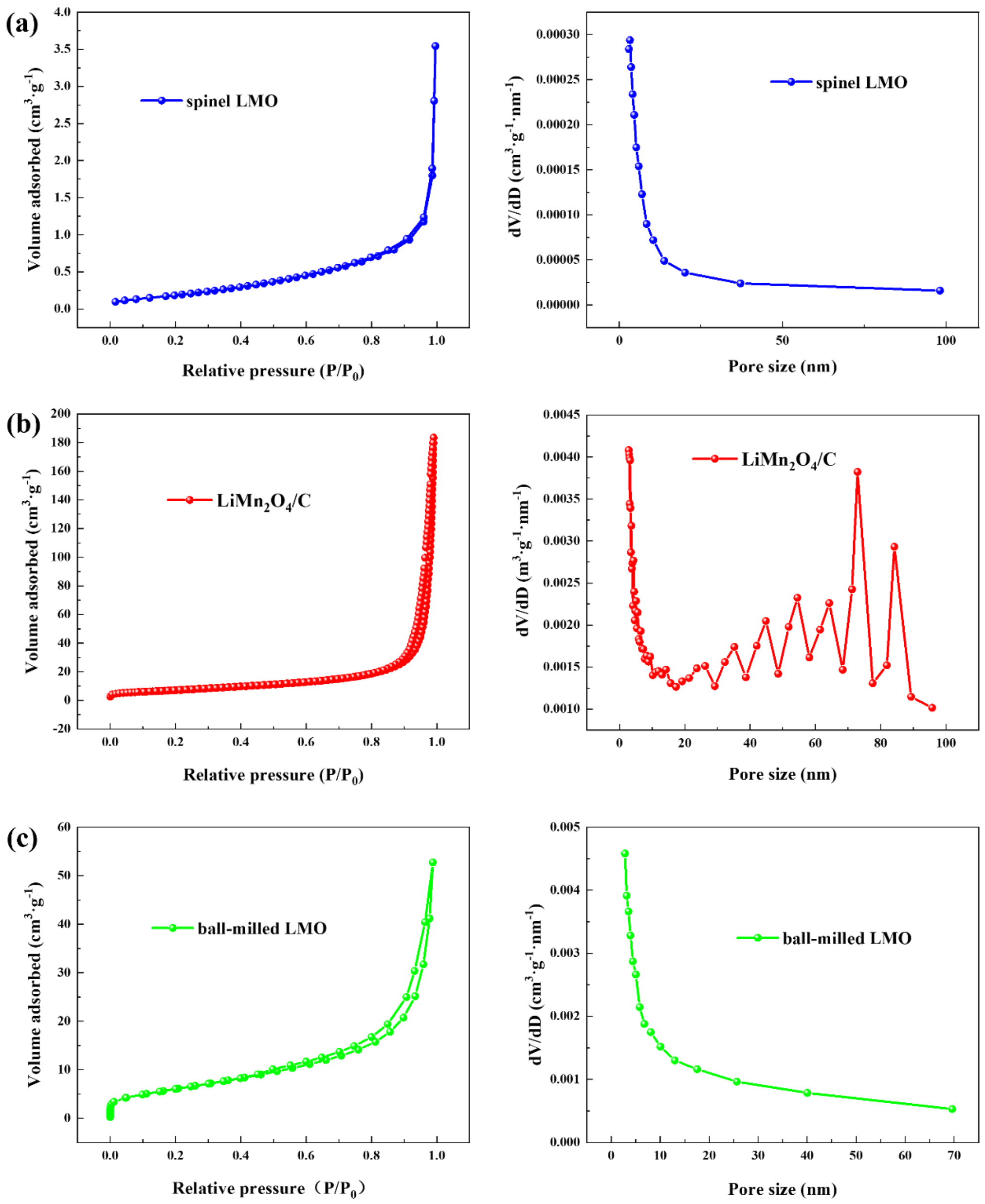

Fig. S21 N_2 adsorption-desorption isotherms and pore sized distributions of (**a**) original LMO, (**b**) $LiMn_2O_4$/C, and (**c**) $LiMn_2O_4$ after 6 hours of ball-milling

Table S1 Comparison of the intensity ratio values of (111) and other XRD peaks after different time lengths of ball-milling with carbon

Ball-milling time	$I_{(111)}/I_{(311)}$	$I_{(111)}/I_{(400)}$	$I_{(111)}/I_{(440)}$
0 hours	1.94	1.80	3.76
2 hours	2.27	2.34	5.26
4 hours	3.21	3.41	6.61
6 hours	3.38	3.77	7.75

Table S2 Comparison of desalination performances of different battery electrode materials for deionization

Cathode ‖ Anode	Applied voltage	Salt concentration	Desalination capacity	References
$Na_2FeP_2O_7$/C ‖ AC	1.2 V	100 mM	32.6 mg·g^{-1}	[S1]
$Na_4Mn_9O_{18}$ ‖ AC	1.2 V	50 mM	31.2 mg·g^{-1}	[S2]
Porous $Ti_3C_2T_x$ ‖ Porous $Ti_3C_2T_x$	1.2 V	171.12 mM	45 mg·g^{-1}	[S3]
$FePO_4$/rGO ‖ rGO	1.8 V	40 mM	85.94 mg·g^{-1}	[S4]
MoS_2 ‖ AC	1.2 V	400 mM	8.81 mg·g^{-1}	[S5]
$Na_4Ti_9O_{20}$/N-doped carbon ‖ Ag/rGO	1.4 V	25.67 mM	62.3 mg·g^{-1}	[S6]
$Na_4Mn_{14}O_{27}$ ‖ $Na_4Mn_{14}O_{27}$	1.0 V	500 mM	76.6 mg·g^{-1}	[S7]
ZIF-67 ‖ ZIF-67	1.2 V	40 mM	21.3 mg·g^{-1}	[S8]
Ag/rGO ‖ $Na_{1.1}V_3O_{7.9}$/rGO	1.4 V	34.22 mM	82.2 mg·g^{-1}	[S9]
$LiMn_2O_4$/C ‖ AC	1.0 V	20 mM	117.3 mg·g^{-1}	this work

Supplementary References

[S1] S. Kim, J. Lee, C. Kim, J. Yoon, $Na_2FeP_2O_7$ as a novel material for hybrid capacitive deionization. Electrochim. Acta **203**, 265-271 (2016). https://doi.org/10.1016/j.electacta.2016.04.056

[S2] J. Lee, S. Kim, C. Kim, J. Yoon, Hybrid capacitive deionization to enhance the desalination performance of capacitive techniques. Energy Environ. Sci. **7**(11), 3683-3689 (2014). https://doi.org/10.1039/C4EE02378A

[S3] W.Z. Bao, X. Tang, X. Guo, S. Choi, C.Y. Wang et al., Porous cryo-dried MXene for efficient capacitive deionization. Joule **2**(4), 778-787 (2018). https://doi.org/10.1016/j.joule.2018.02.018

[S4] J. Ma, L. Wang, F. Yu, X.H. Dai, Mesoporous amorphous $FePO_4$ nanosphere@graphene as a faradic electrode in capacitive deionization for high-capacity and fast removal of NaCl from water. Chem. Eng. J. **370**, 938-943 (2019). https://doi.org/10.1016/j.cej.2019.03.243

[S5] F. Xing, T. Li, J.Y. Li, H.R. Zhu, N. Wang et al., Chemically exfoliated MoS_2 for capacitive deionization of saline water. Nano Energy **31**, 590-595 (2017). https://doi.org/10.1016/j.nanoen.2016.12.012

[S6] Y.Y. Zhang, K. Yang, H.B. Yu, W.J. Shan, Z.N. Lou et al., Boosting deionization capability by effectively improving sodium-ion storage capacity based on robust interfacial electronic interaction within 3D $Na_4Ti_9O_{20}$/N-doped porous carbon heterostructures. J. Mater. Chem. A **9**(43), 24374-24386 (2021). https://doi.org/10.1039/d1ta06369c

[S7] Y.B. Zhao, A. Gong, Y. Liu, K.X. Li, Facile synthesis and enhanced desalination performance of a novel layered $Na_4Mn_{14}O_{27}$ made from earth-abundant element in

capacitive deionization. Sep. Purif. Technol. **258**, 118057 (2021). https://doi.org/10.1016/j.seppur.2020.118057

[S8] Y.Q. Zhang, L. Ji, Y. Zheng, H.W. Liu, X.T. Xu, Nanopatterned metal-organic framework electrodes with improved capacitive deionization properties for highly efficient water desalination. Sep. Purif. Technol. **234**, 116124 (2020). https://doi.org/10.1016/j.seppur.2019.116124

[S9] Z.S. Yue, Y.L. Ma, J.W. Zhang, H. Li, Pseudo-capacitive behavior induced dual-ion hybrid deionization system based on Ag@rGO parallel to $Na_{1.1}V_3O_{7.9}$@rGO. J. Mater. Chem. A **7**(28), 16892-16901 (2019). https://doi.org/10.1039/c9ta03570b
